# Pharmacological Activity of Flavonoid Quercetin and Its Therapeutic Potential in Testicular Injury

**DOI:** 10.3390/nu15092231

**Published:** 2023-05-08

**Authors:** Xiaohui Zhang, Yufeng Tang, Guangping Lu, Junlian Gu

**Affiliations:** 1 School of Nursing and Rehabilitation, Cheeloo College of Medicine, Shandong University, Jinan 250012, China; 202116672@mail.sdu.edu.cn (X.Z.); 202020820@mail.sdu.edu.cn (G.L.); 2 Department of Orthopedic Surgery, The First Affiliated Hospital of Shandong First Medical University, Jinan 250014, China; ttang1987@163.com

**Keywords:** quercetin, testicular injury, antioxidant, anti-apoptosis, anti-inflammatory

## Abstract

Quercetin is a natural flavonoid widely found in natural fruits and vegetables. Recent studies have shown that quercetin mediates multiple beneficial effects in a variety of organ damage and diseases, and is considered a healthcare supplement with health-promoting potential. Male infertility is a major health concern, and testicular damage from multiple causes is an important etiology. Previous studies have shown that quercetin has a protective effect on reproductive function. This may be related to the antioxidant, anti-inflammatory, and anti-apoptotic biological activities of quercetin. Therefore, this paper reviews the mechanisms by which quercetin exerts its pharmacological activity and its role in testicular damage induced by various etiologies. In addition, this paper compiles the application of quercetin in clinical trials, demonstrating its practical effects in regulating blood pressure and inhibiting cellular senescence in human patients. However, more in-depth experimental studies and clinical trials are needed to confirm the true value of quercetin for the prevention and protection against testicular injury.

## 1. Introduction

Polyphenolic compounds, also known as polyphenols, are the collective name for all phenolic derivatives. They share the same structural characteristics, namely the presence of one or more six-carbon aromatic rings and two or more phenolic hydroxyl groups [1]. Plant polyphenols, also known as plant tannins, are natural compounds found mainly in plants and often have potential health benefits. Over 8000 polyphenols have been identified and divided into four main groups: flavonoids, stilbenes, lignans, and phenolic acids [2]. Among these, flavonoids are secondary metabolites of polyphenols, usually yellow pigments, that make up about two-thirds of the polyphenols in the human diet, and they are phytochemicals that humans cannot synthesize [3]. Flavonoids are a series of C6-C3-C6 compounds consisting of two benzene rings (A-ring and B-ring) linked by a central three-carbon bond. According to the degree of oxidation of carbon bonds and the differences in B-ring attachment sites, flavonoids can be divided into subgroups such as flavonoids, flavonols, flavanols, catechins, isoflavones, and anthocyanins [3,4]. In recent years, phytochemicals, especially dietary polyphenols and flavonoids, have received increasing attention because of their beneficial effects on human health, and have become an emerging research hotspot in nutrition. Numerous studies have demonstrated the therapeutic potential and health-promoting effects of flavonoids on obesity and diabetes [5,6], cardiovascular diseases [7], cancer [8], neurodegenerative diseases [9], autoimmune diseases [10], and a variety of tissue and organ damage (e.g., gastrointestinal tract [11], kidney [12], liver [13], testes [14]).

Male infertility is a major health problem worldwide, and can cause severe psychological distress and financial burden to patients. Clinical and experimental studies have shown that a variety of diseases and risk factors can contribute to male infertility, which can be characterized as congenital, acquired, and idiopathic risk factors. Among them, congenital factors mainly include genetic and developmental perturbations such as Klinefelter syndrome, anorchia, cryptorchidism, and congenital absence of vas deferens; acquired factors include germ cell tumors, urogenital injuries from various causes (e.g., varicocele and testicular torsion), and systemic diseases (live cirrhosis, renal failure); idiopathic factors, in contrast, mainly refer to causes unrelated to male factors, such as endocrine disturbances, unhealthy lifestyles (smoking, alcohol consumption, obesity), psychological factors, and environmental pollutant exposure [15,16]. In addition to the above classification, there is another type of unexplained male infertility, which refers to unexplained infertility with normal seminal parameters and normal ovulation and fallopian tube patency of the female partner [17]. The above types of etiology directly or indirectly cause testicular injury, resulting in spermatogenic dysfunction—even non-obstructive azoospermia—or lead to blockages of the vas deferens in which testicular spermatogenesis is normal, but sperm cannot be discharged from the body, which is known as obstructive azoospermia, both of which are important causes of male infertility. Research has shown that many plant-derived compounds and nutrients, such as flavonoids, resveratrol, sulforaphane, and curcumin, have great potential to prevent and improve organ and tissue damage, reduce the incidence of cancer, mitigate inflammatory responses, and promote health. Among these, the flavonoid quercetin has been shown to improve testicular damage caused by a variety of etiologies. Therefore, the purpose of this review article is to summarize the physiological functions of quercetin, to assess the experimental evidence for its protective role in various types of testicular injury, and to summarize the current status of its clinical studies.

## 2. Quercetin

**Figure 1:**
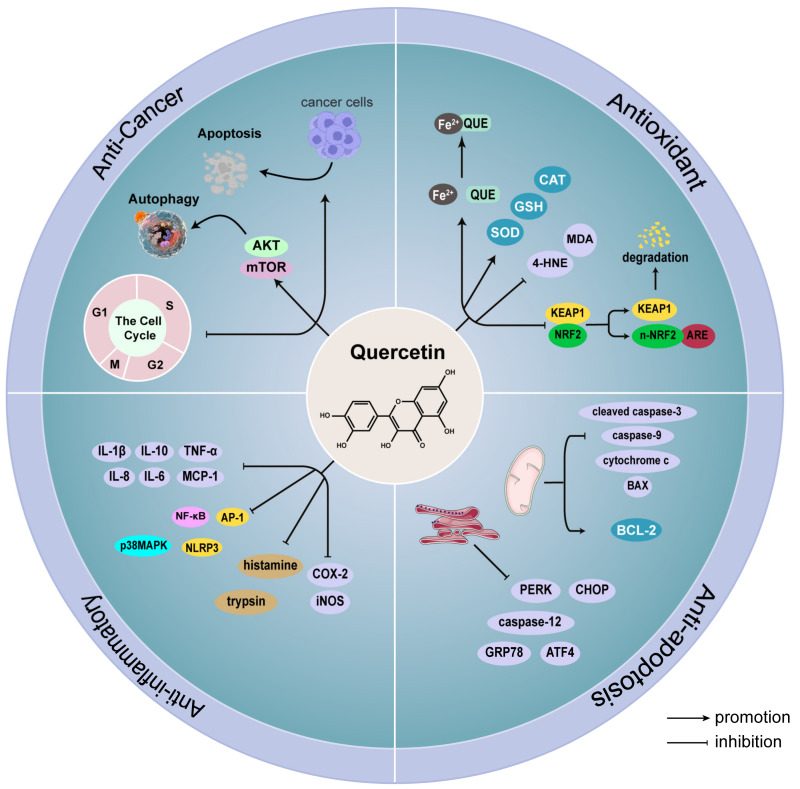
The main biological activities of quercetin and its mechanism. Abbreviations: QUE, quercetin; SOD, superoxide dismutase; CAT, catalase; GSH, reduced glutathione; 4-HNE, 4-hydroxynonenal; MDA, malondialdehyde; KEAP1, Kelch-like ECH-associated protein 1; NRF2, nuclear factor erythroid 2-related factor 2; ARE, antioxidant response element; BCL-2, B-cell lymphoma-2; BAX, BCL-2 associated X; PERK, protein kinase RNA-like ER kinase; CHOP, CCAAT/enhancer-binding protein homologous protein; GRP78, glucose-regulated protein 78; ATF4, activating transcription factor 4; TNF-α, tumor necrosis factor-α; MCP-1, monocyte chemotactic protein-1; IL-10, interleukin-10; IL-6, interleukin-6; IL-1β, interleukin-1β; IL-8, interleukin-8; AP-1, activator protein 1; NF-κB, nuclear factor kappa-B; p38PAPK, p38 mitogen-activated protein kinase; NLRP3, NOD-like receptor thermal protein domain associated protein 3; COX-2, cyclooxygenase-2; iNOS, inducible nitric oxide synthase.

The polyphenol quercetin (3,3′,4′,5,7-pentahydroxyflavone) is a dietary flavonoid and is classified as a flavonol, one of the subclasses of flavonoid compounds [18]. It may also be part of other flavonoids such as hesperidin, naringenin, and rutin [19]. As secondary metabolites of plants, flavonoids are widely distributed in nature. Quercetin is found in many foods such as apples, cranberries, red onions, grapes, cherries, broccoli, peppers, camellias, citrus fruits, asparagus, and radishes [19,20]. The molecular formula of quercetin is C_15_H_10_O_7_ and its structure contains two benzene rings and five hydroxyl groups [18,21]. Quercetin usually exists as an O-glycoside and at least one hydroxyl group in its structure is replaced by various types of sugars, resulting in derivatives such as quercetin, isoquercitrin, hyperoside, and rutin [22]. Quercetin and its derivatives are usually yellow powders with poor water solubility and their stability is affected by oxygen, temperature, pH, the concentration of other antioxidants [21], etc. Quercetin has been shown to possess a variety of biological activities, including antioxidant, anti-inflammatory, anti-apoptotic, anti-cancer, anti-aging, immunomodulatory, anti-viral, and anti-allergy [22,23,24]. The mechanism by which quercetin exerts its main biological activity is shown in Figure 1.

### 2.1. Antioxidant Effect of Quercetin

The imbalance between oxidative and antioxidant capacity in the organism leads to oxidative stress, which tends to oxidize and cause excessive production of highly reactive molecules, such as reactive oxygen radicals (ROS) and reactive nitrogen radicals (RNS), leading to tissue damage. ROS include hydroxyl radicals (OH^−^), hydrogen peroxide (H_2_O_2_), superoxide anion (O^2−^), and nitric oxide (NO), which are highly unstable. Therefore, scavenging oxygen-containing radicals is an important process of antioxidants. Quercetin is one of the dietary oxidants that enhances the antioxidant defense system and eliminates oxygen radicals generated during cellular metabolism. The antioxidant action of quercetin involves several mechanisms. On the one hand, as a polyphenol, the polyphenol hydroxyl group in its molecular structure can act as a hydrogen donor and react with free radicals to directly scavenge ROS [25,26]; on the other hand, quercetin can inhibit the activity of enzymes involved in free radical production, thereby inhibiting ROS production [25,27]. In addition, quercetin can increase the levels of antioxidant enzymes (such as superoxide dismutase (SOD), catalase (CAT), etc.) and reduced glutathione (GSH) [25,28], and decrease the levels of malondialdehyde (MDA) and 4-hydroxynonenal (4-HNE) [29] to maintain a stable redox state in cells. It has also been shown that quercetin is an inhibitor of protein–protein interactions between Kelch-like ECH-associated protein 1 (KEAP1) and nuclear factor erythroid 2-related factor 2 (NRF2) [30], mediating the degradation of KEAP1 protein, improving the nuclear translocation of NRF2 and its binding activity, and enhancing the binding of NRF2 to the antioxidant response element (ARE) [25]. Quercetin can also regulate the expression of the downstream antioxidant enzyme thioredoxin (Trx) system of NRF2 by increasing the expression of Trx at the mRNA and protein levels, inhibiting the expression of intracellular Trx-interacting protein (Txnip) [31], and enhancing the antioxidant defense capacity of the body. In addition, quercetin induces the chelation of ROS-producing metal ions and attenuates oxidative damage due to iron overload [26].

### 2.2. Anti-Apoptotic Effect of Quercetin

Apoptosis, a process of programmed cell death, is an integral component of various cellular processes that maintain intracellular homeostasis. However, insufficient or excessive apoptosis can have adverse effects on cells and tissues. Apoptosis includes: intrinsic pathway, i.e., mitochondria-mediated apoptosis pathway; extrinsic pathway, i.e., death receptor-mediated apoptosis pathway; and endoplasmic reticulum stress (ERS)-mediated apoptosis pathway. Numerous studies have shown that quercetin can influence apoptosis by modulating the above pathways. In fact, the anti-apoptotic effect of quercetin is inextricably linked to the antioxidant effect. Excess ROS generated by oxidative stress is one of the main causes of apoptosis. Mitochondria are the main site of ROS production and the primary target of ROS. In vitro cell models have demonstrated that quercetin enhances antioxidant capacity by activating P38 signaling to promote NRF2 and its downstream heme oxygenase-1 (HO-1) protein expression, and increases the activity of antioxidant enzymes, thereby reducing ROS production, rescuing mitochondrial membrane potential, and mitigating mitochondrial damage [32,33]; it also reduces caspase-9, cleaved caspase-3, cytochrome C, cleaved Poly(ADP-ribose) polymerase 1 (PARP1) activity, and BCL-2 associated X protein (BAX)/B-cell lymphoma-2 (BCL-2) ratio to inhibit mitochondrial pathway apoptosis [32,34,35]. It can also play a beneficial role in apoptosis by inhibiting Phosphatase and tensin homologue deleted on chromosome ten (PTEN) expression and enhancing PI3K/AKT phosphorylation [34]. In ERS-induced apoptosis, quercetin can reduce glucose-regulated protein 78 (GRP78), protein kinase RNA-like ER kinase (PERK), eukaryotic translation initiation factor 2A (eIF2α), activating transcription factor 4 (ATF4), CCAAT/enhancer-binding protein homologous protein (CHOP), inositol-requiring enzyme-1 (IRE1), X-box binding protein 1 (XBP1), and ATF6 expression at the mRNA [36,37] and protein levels, and reduce the activation of caspase-12 [36,37], a protein characteristic of ERS-induced apoptosis; these results suggest that quercetin could also play a role in attenuating ERS-induced apoptosis. As for death receptor pathway-induced apoptosis, although there are no studies related to quercetin, Liu et al. showed that quercetin-3-O-galactoside, a derivative of quercetin, was found to decrease the protein expression of Fas, Fas ligand (Fas-L) in EA.hy926 cells in a dose-dependent manner, suggesting that quercetin may also have this effect, but this remains to be clearly verified by experiments [38]. In addition to the above mechanisms, the nuclear factor kappa-B (NF-κB) signaling pathway responds to a variety of deleterious components (e.g., free radicals, cytokines, bacterial toxins, and viruses) that can contribute to inflammation, apoptosis, and cancer gene expression. In hepatocytes, quercetin was shown to ameliorate oxidative damage by inhibiting p65 NF-κB expression [39].

### 2.3. Anti-Inflammatory Effect of Quercetin

Inflammation is an adaptive response that underlies a variety of physiological and pathological processes triggered by noxious condition. Many studies have shown that quercetin can provide potent anti-inflammatory effects through inhibiting inflammatory factors and inflammatory signaling pathways. Quercetin has been shown to inhibit the expression of proinflammatory cytokines (tumor necrosis factor-α (TNF-α), interleukin (IL)-6, monocyte chemotactic protein-1 (MCP-1), IL-10, etc.) in lipopolysaccharide-stimulated neutrophils [40], macrophages [41], and hepatictissue from tripterygium-induced liver injury [42] and epididymal adipose tissue from high-fat-fed mice [43]. In addition, quercetin decreased the number of M1 macrophages and increased the number of M2 macrophages in epididymal adipose tissue [43], and decreased the gene expression of cyclooxygenase-2 (COX-2) and inducible nitric oxide synthase (iNOS) [41], which are enzymes involved in the inflammatory response. Quercetin also inhibited the activation of transcription factors NF-κB and activator protein 1 (AP-1) in TNF-α-treated human umbilical vein endothelial cells [44]. Furthermore, in diabetic nephropathy rats, quercetin reduced renal inflammation by decreasing NOD-like receptor thermal protein domain associated protein 3 (NLRP3) activation [45]. Multiple studies have shown that mast cells are involved in the pathogenesis of several inflammatory diseases. Activated mast cells produce inflammatory and chemotactic factors such as TNF-α, IL-1β, IL-6, IL-8, IL-4, IL-13, and transforming growth factor-β (TGF-β) [46]. In addition, quercetin has numerous effects on reducing mast cell recruitment [43], maintaining mast cells stability as well as inhibiting the release of mast cell-like trypsin and histamine, which may be related to the inhibition of intracellular calcium influx and calcium-insensitive protein kinase C theta (PKCθ) [47]; meanwhile, quercetin blocked the activation of p38 mitogen-activated protein kinase (p38MAPK) and NF-κB in mast cells, thereby attenuating the expression of pro-inflammatory cytokines such as TNF-α, IL-6β, IL-8, and IL-1 [46].

### 2.4. Anti-Cancer

Cancer is a serious threat to human health with high morbidity and mortality rates worldwide. The treatment of cancer usually involves radiotherapy, surgery, and chemotherapy. However, the use of chemotherapeutic drugs is hampered by their side effects and resistance; therefore, it is an important task to find other drugs that have good anti-cancer effects but low side effects, which are expected to be used in combination with chemotherapy drugs to fully realize anticancer efficacy. As research on quercetin continues, many researchers have found that quercetin produces anticancer effects against a wide range of tumors under both in vivo and in vitro conditions. The anticancer effect of quercetin is closely related to its regulation of apoptosis. Quercetin can promote tumor cell apoptosis through both intrinsic and extrinsic apoptotic pathways. Quercetin can: directly bind to the BH3 structural domain of BCL-2 and B-cell lymphoma-extra large (BCL-XL)proteins [48], thereby inhibiting their anti-apoptotic activity; stimulate the expression of pro-apoptotic genes such as BAX, BCL-2 associated agonist of cell death (BAD), and apoptotic protease activating factor 1(Apaf-1) [49]; increase the release of cytochrome C from the mitochondria into the cytoplasm [50]; and promote the activation of caspase-9 and caspase-3 [50,51,52] from the mitochondria-induced apoptosis pathway to induce apoptosis in tumor cells. Meanwhile, quercetin increased the expression of tumor necrosis factor-related apoptosis-inducing ligand (TRAIL), caspase-8, Fas, Fas-L, and Fas-associated protein with death domain (FADD), which enhanced apoptosis in the death receptor pathway [53]. In addition to inducing apoptosis in cancer cells, many studies have shown that quercetin can also exert a fundamental role for cell cycle regulation in the chemosensitivity of cancer cells. Quercetin down-regulates the expression of cyclin D, cyclin E, E2F1, E2F2, and cell cycle protein-dependent kinase 1 (CDK1), and induces p21 expression in a checkpoint kinase 2 (CHK2)-dependent manner [54,55], leading to cell cycle arrest in G1 and G2/M phases in human breast cancer cell lines (SKBR3 cells and MDA-MB-453 cells) and human leukemia U937 cells; moreover, in human liver cancer cells (HepG2), quercetin-3-O-glucoside was found to inhibit DNA topoisomerase II activity, affecting DNA replication and transcription, and increase S-phase cell populations and cell cycle arrest, ultimately leading to cell death [56].

Autophagy also plays a complex role in tumorigenesis. On the one hand, autophagy can enhance the tolerance of tumor cells to stress and maintain their survival in an unfavorable environment, and the nutrition provided by cellular autophagy can promote tumor growth; on the other hand, autophagy can inhibit tumorigenesis and metastasis at various stages of tumor development, and even act as a death pathway for tumor cells when primary apoptosis is defective. The different outcomes of autophagy production in tumor cells make the role of autophagy drugs for anticancer therapy uncertain. However, it is worth mentioning that many studies have shown that quercetin can regulate autophagy through various mechanisms and thus inhibit cancer progression. The main mode of glucose metabolism in tumor cells is glycolysis, also known as the “Warburg effect”. While inhibiting the Akt-mTOR pathway [57,58,59] and thus promoting autophagy, quercetin can also downregulate the expression of glucose transporter protein 1 (GLUT1) and the expression of pyruvate kinase M2 (PKM2) and lactate dehydrogenase A (LDHA), which are key enzymes of glycolysis, and inhibit tumor cell glycolysis and cell migration [58].

## 3. Protective Effect of Quercetin on Testicular Injury

Decreased male fertility due to structural damage and dysfunction of the testis has been an unavoidable focus of research in the field of reproductive medicine. The testis is a male reproductive organ whose structure includes the seminiferous tubules and their surrounding connective tissue, and whose main functions are to produce sperm and secrete androgens. The spermatogenic cell includes spermatogonia, primary spermatocytes, secondary spermatocytes, spermatids, and spermatozoa, and they are sequentially arranged from the basement membrane to the lumen to form the epithelium of the seminiferous tubules, also known as the spermatogenic epithelium, where spermatozoon eventually enter the lumen of the seminiferous tubules after molding. In addition to spermatogenic cells, the seminiferous tubules also contain the sustentacular cell (also known as a Sertoli cell) that performs various functions such as nutrition, support, protection, and transport. In the mammalian testis, there is also a blood–testis barrier (BTB) provided by Sertoli cells near the basal membrane of the spermatogenic epithelium and composed of tight junction (TJ), testis-specific atypical cell-cell adherens junction (the basal ectoplasmic specialization (ES)), desmosome-like junction, and gap junction (GJ), which form an immune barrier and create a favorable microenvironment for sperm production [60]. In the interstitial space of the seminiferous tubules, there are clusters of Leydig cells that secrete most of the androgens, with testosterone being the major androgen. The endocrine regulation of the testis is also reflected in the maintenance of stable androgen levels through negative feedback regulation of the hypothalamic–pituitary–testicular (HPT) axis.

In the past decade, numerous studies have confirmed that many congenital or pathological causes of testicular damage and impaired spermatogenesis contribute to male infertility, and quercetin has been reported to play a protective role in testicular damage caused by a variety of etiologies, such as chemotherapeutic drugs, heavy metal exposure, environmental pollutants, and diabetes mellitus.

### 3.1. Diabetes

Studies have shown that obesity and diabetes mellitus type 2 (T2DM) are closely associated with low testosterone levels, and in particular, hyperglycemia and impaired glucose regulation caused by T2DM are among the causes of male infertility [61]. Experiments in diabetic rats have shown that the effects of diabetes on reproductive function include dysregulation of steroid production such as androgens, abnormal spermatogenesis, and sexual dysfunction, which may be caused by the following mechanisms: the expression of enzymes required for normal spermatogenesis, such as cytochrome P450 family 11 subfamily A member 1 (CYP11A1), cytochrome P450 family 17 subfamily A member 1 (CYP17A1), steroidogenic acute regulatory (StAR) protein, 3β-hydroxysteroid dehydrogenase (3β-HSD), and 17β-HSD decreased; impairment of seminiferous tubules morphology and testicular mesenchymal structure; detrimental effects on HPT axis function; aberrant DNA repair; and impaired antioxidant function [62,63]. Quercetin has multiple beneficial effects on diabetes-induced testicular damage, with antioxidant stress, anti-apoptosis, and anti-inflammatory being the main pathways of action. Diabetes-induced decreases in testicular total antioxidant capacity (TAC), SOD, and CAT and elevated MDA were all attenuated by 20 mg/kg/day quercetin treatment for six weeks in Zucker Diabetic Fatty rats [64]. Upregulation of the pro-apoptotic proteins BAX due to prolonged hyperglycemia, and *caspase-3* mRNA expression, the executioner of apoptosis, downregulation of the anti-apoptotic protein BCL-2, were also significantly improved by quercetin [63,64]. After 20 mg/kg/day quercetin treatment for eight weeks in Wistar albino rats, a decrease in the number of terminal dUTP nick end-labeling (TUNEL)-positive cells and an increase in the expression of proliferating cell nuclear antigen (PCNA) in testis all suggest an improvement in DNA synthesis [65]. Notably, quercetin also appears to have an effect on the pancreatic islets of diabetic rats, and a study by Tiss et al. showed that a methanolic extract of Globularia alypum (containing several chemicals, including quercetin) ameliorated pancreatic β-cell damage and death in diabetic Wistar rats, which was able to increase insulin secretion and maintain better blood glucose levels, thus somewhat reducing testicular damage caused by the hyperglycemic environment of the body. It can also improve the process of spermatogenesis and increase the number of spermatozoon in the seminiferous tubules to the level of normal rats [66].

### 3.2. Environmental Pollutants

#### 3.2.1. Heavy Metals Exposure

In nature, heavy metals and chemical pollutants are exposed to the environment, and these substances are often highly toxic, and human exposure to them directly or indirectly through water, air, soil, or food intake can cause irreversible negative effects on the health of the organism. In particular, these toxic substances are difficult to be excreted, and their toxicity will accumulate in the body, causing gradually increasing damage that can lead to cancer, multi-organ damage, and even increased mortality. Numerous previous studies have confirmed that heavy metal pollution has a significant impact on the male reproductive system and is one of the major causes of human infertility [67]. Possible effects of different heavy metals on testicular function include induction of apoptosis in germ cells [68] and Sertoli cells [69], disruption of the structure of the seminiferous tubules and the BTB, impairment of spermatogenesis [67,70], exacerbation of oxidative stress [68,71,72,73] and ERS, induction of Leydig cell tumors [67], and disruption of potassium and calcium channels, which are involved in acrosome reactions [74].

Lead (Pb) is a toxic and widespread environmental contaminant. One of the major mechanisms of Pb-induced toxicity is oxidative stress, and studies have shown that quercetin, a natural antioxidant, protects testis from Pb-induced oxidative stress and apoptosis [71,75], and as a metal chelator, quercetin can form insoluble complexes with Pb, thereby reducing its toxicity. It also has ameliorative effects on weight loss, decreased sperm quality and androgen levels induced by Pb acetate (PbAc) exposure [71]. However, there are conflicting results regarding the protective effects of quercetin against Pb-induced testicular damage. Claudin 11 and ocludin are tight junction proteins in BTB, and connexin 43 (Cx43) is a gap junction protein, and Dolati et al. showed that the expression of claudin 11, ocludin, and Cx43 were all affected by Pb exposure, while serum testosterone levels did not change significantly after PbAc combined with quercetin treatment; no significant improvement in the expression of claudin 11 and ocludin was found, indicating that quercetin did not improve the BTB [76].

**Figure 2:**
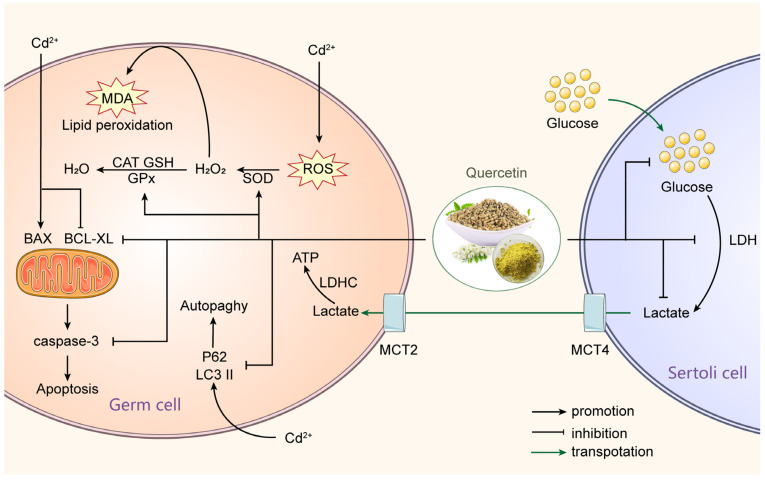
The protective mechanism of quercetin against Cd-induced testicular injury. Quercetin reduces Cd-induced oxidative stress by increasing SOD, CAT, GSH, glutathione peroxidase (GPx) expression, and reducing malondialdehyde and H_2_O_2_ production; it improves apoptosis mainly by promoting B-cell lymphoma-extra large (BCL-XL) expression and inhibiting BAX and caspase-3 expression; it inhibits autophagy by reducing P62 and LC3-II; it also has an effect on testicular energy metabolism, mainly by inhibiting high glucose, high lactate, and high lactate dehydrogenase (LDH) caused by germ cell compensation.

Cadmium (Cd) is a silvery-white, highly toxic heavy metal. It is one of the most common environmental and industrial pollutants. In the pathogenesis of Cd-induced testicular damage, oxidative stress-mediated testicular toxicity is the main cause. Cd can lead to excessive free radical production and induce lipid peroxidation, alter the activity of endogenous antioxidant enzymes, and ultimately threaten male fertility [77]. Quercetin may act by reducing oxidative stress and restoring antioxidant capacity, as evidenced by increased levels of GSH, glutathione peroxidase (GPx), CAT, vitamin C, vitamin E, TAC, and decreased MDA and H_2_O_2_ production in the testicular homogenate [68,72,73]. There is no doubt that quercetin also has a protective effect against apoptosis through increasing the expression of BCL-XL and decreasing the expression of BAX and caspase-3 [73]. In addition, quercetin reduces Cd-induced autophagy in testis cells. Wang et al. showed that the levels of P62 and LC3-II proteins increased after Cd treatment, indicating an increase in autophagy in testicular cells, and quercetin treatment restored autophagy to normal levels [78]. Quercetin also attenuated the inhibitory effect of Cd on steroidogenesis, penile erection, and decreased sexual behavior [79]. Notably, quercetin also affects the energy metabolism of the testes. Studies have shown that germ cells depend on lactate for energy. The process begins with the conversion of glucose to lactate in Sertoli cells, which is catalyzed by lactate dehydrogenase (LDH). Lactate then leaves Sertoli cells via the monocarboxylate transporter 4 (MCT4) and enters the germ cells via MCT2. ATP is produced in germ cells through the action of LDHC. Nna et al. showed that the testes were in high glucose, high lactate, and high LDH activity with cadmium chloride (CdCl_2_) administration, the reason for which may be a compensatory mechanism of the germ cells, and basically returned to normal levels after quercetin treatment [68]. The protective mechanism of quercetin against Cd-induced testicular injury is shown in Figure 2.

Manganese (Mn) is an essential trace element for the human body, and low concentrations are essential for biological processes such as manganese metabolism, but excessive Mn exposure can lead to neurotoxicity, reproductive toxicity, and other effects. Adedara et al. showed that Mn induced functional alterations in the hypothalamic–pituitary–testicular axis of rats in rat model experiments, and that quercetin increased antioxidant enzyme activity, reduced the expression of inflammatory biomarkers, and maintained stable levels of hormones associated with the gonadal axis [80].

Quercetin may also play a critical role in preventing and ameliorating testicular toxicity produced by zinc oxide nanoparticles (ZnONPs) [81] and titanium dioxide nanoparticles (NTiO_2_) [82].

#### 3.2.2. Other Environmental Pollutants Exposure

In addition to heavy metals, there are many other environmental contaminants that cause testicular toxicity, such as pesticides, herbicides, diesel exhaust particles, plastic products, plasticizers, etc. Atrazine (2-chloro-4-ethylamino-6-isopropyl-amino-s-triazine) is a widely used pesticide worldwide and one of the most common contaminants in soil and water that persists in the environment for long periods. The reproductive toxicity of atrazine is mainly manifested by interfered testicular Leydig cell function and viability [83,84], increased levels of oxidative stress and lipid peroxidation [69,84,85,86], and interference with steroid gene expression under in vivo or in vitro conditions [83,84,85]. Atrazine has also been shown to cause DNA fragmentation, indicating an increase in apoptosis [85]. Lauritta et al. also showed that TNF-α and myeloperoxidase (MPO) were also elevated in atrazine-treated rats [86], indicating an increased inflammatory response. Numerous studies have shown that quercetin treatment is good at eliminating the above damage and improving reproductive function. Arsenic (As) is a toxic environmental pollutant used in the production of pesticides, glass, fireworks, etc. Quercetin also significantly ameliorated sperm loss and testicular pathological damage induced by As exposure. PCNA, a marker of cell proliferation, was expressed in spermatogonia of all stages and early spermatocytes in the seminiferous tubules [87,88]. As treatment significantly reduced the number of PCNA-positive cells in testicular tissue, whereas quercetin increased the PCNA index [88]. The environmental pollutant Bisphenol A (BPA) is a well-known xenoestrogen that causes endocrine disruption in the male reproductive organs by mimicking estrogenic activity and inducing hormonal imbalance in rats. Molecular dynamics simulation experiments by Samova et al. found that BPA can bind to steroid-binding proteins to reduce their activity, whereas quercetin can compete with BPA, and this seems to be a possible mechanism by which quercetin reduces the testicular toxicity of BPA [89]. In addition to these effects, quercetin has been shown in numerous studies to be protective against testicular damage induced by other environmental pollutants such as crude oil vapor (COV) [90], diesel exhaust particles (DEPs) [91], polychlorinated biphenyls (PCBs) [92], carbon tetrachloride (CCl4) [93], 4-nitrophenol (PNP) [94], 2,3,7,8-tetrachlorodibenzo-p-dioxin (TCDD) [95], acetylene [96], and phthalates (PEs) [97,98]. The protective effects of quercetin on exposure to different environmental pollutants are described in Table 1.

### 3.3. Drugs

Studies have shown that though many drugs have a therapeutic effect on diseases, they also cause unavoidable damage to various organs, and reproductive toxicity is one of the most common side effects. The use of chemotherapeutic drugs is one of the etiologies of testicular damage. Doxorubicin (DOX) is an anthracycline antibiotic, and it is particularly important to note that DOX has a dose-dependent and cumulative toxicity, with the greater the amount accumulated in the body, the more toxic the side effects. The mechanism by which DOX causes testicular toxicity is not fully understood and is usually thought to be related to oxidative stress and cell cycle inhibition. Studies have found that oral 80 mg/kg quercetin alone or in combination with 10 mg/kg sitagliptin for 21 days is effective in ameliorating testicular damage in Wistar rats, and the combination may even restore damage to control levels [99]. Cyclophosphamide (CYP) is a widely used antitumor and immunosuppressive drug that causes multi-organ toxicity in humans and experimental animals. In addition to inducing dense oxidative stress, studies have shown that CYP exacerbates the inflammatory response and increases serum levels of IL-6 and interferon gamma (INF-γ) in the testis. Indoleamine 2,3-dioxygenases (IDO) and tryptophan 2,3-dioxygenase (TDO) are rate-limiting enzymes of tryptophan metabolism; tryptophan degradation produces kynurenine, which has been shown to drive inflammation and participate in immune regulation. CYP increases the activity of both in the testis, while oral 50 mg/kg quercetin for 7 days inhibits their activity to protect testicular integrity in Wistar rats [100]. Besides the drugs mentioned above, quercetin protects against reproductive toxicity caused by cisplatin [101,102], docetaxel [103], letrozole [104], triptolide [105], and sulfasalazine [106].

### 3.4. Testicular Torsion/Detorsion

Torsion of the testis is an acute urological condition that occurs primarily in male newborns, children, and adolescents [107]. Spermatogonium and spermatocytes are cells that are very sensitive to testicular ischemia, and testicular torsion leads to the obstruction of venous return and arterial blood flow, resulting in an ischemic and hypoxic state of the testicular tissue. Ischemia and reperfusion, especially reperfusion after prolonged ischemia, can lead to excessive production and release of ROS, NO, and cytokines, as well as calcium overload [107,108], which may cause irreparable damage of testis. Antioxidant therapy has been shown to successfully reduce reperfusion injury in several organs and systems. Quercetin has been shown to have antioxidant properties and to increase endothelial NO synthase (eNOS) expression, which significantly improves testicular histopathologic parameters [107,108,109].

## 4. Clinical Studies, Application Prospects, and Limitations of Quercetin

### 4.1. Clinical Trials on Quercetin

Although no clinical trials have been conducted to demonstrate the role of quercetin in human male infertility patients, clinical trials have been reported on the protective effects of quercetin in humans in other diseases. A series of randomized, double-blind, placebo-controlled, crossover studies showed that six weeks of quercetin administration (162 mg/day) in overweight or obese hypertensive patients did not cause side effects such as systemic inflammation and damage to liver and kidney function, and had no significant effect on blood lipids, fasting glucose, and insulin levels, but improved ambulatory blood pressure (ABP) in a subgroup of hypertensive patients [110]; four weeks of high-dose quercetin (730 mg/day) also lowered blood pressure in patients with stage 1 hypertension [111]. This hypotensive effect was more pronounced in overweight obese patients carrying the apolipoprotein (apo) ε3 allele (apo ε3/ε3) or with a high cardiovascular disease (CVD) risk phenotype [112,113]. The effect of quercetin on improving endothelial function is controversial, as supplementation with quercetin-rich onion peel extract (OPE) for 12 weeks improved circulating endothelial progenitor cells (EPC) and flow-mediated endothelium-dependent vasodilation (FMD) [114], whereas Brüll et al. showed no effect of quercetin on vascular endothelial function [110,115]. These discrepancies may be attributed to differences in study protocols, dose, and duration of quercetin administration, and the way endothelial function is assessed.

Senolytics, a combination of senescence and lytic, are a large class of drugs that selectively eliminate senescent cells by interfering with senescent signaling pathways and temporarily disabling the senescent anti-apoptotic pathway (SCAP) [116]. Dasatinib in combination with quercetin is a recent discovery of senolytic molecules with significant anti-aging effects. Idiopathic pulmonary fibrosis (IPF) is a chronic progressive fibrotic lung disease in which cellular senescence is the key mechanism. Justice et al. used senolytics therapy for the first time in a randomized controlled trial in IPF and showed that, despite some limitations, oral 100 mg/day dasatinib and 1250 mg/day quercetin at three-days/week for three weeks had significant beneficial effects on lung function and even bodily function [117]. Senolytics (D + Q) have also shown some improvement in Alzheimer’s disease [118] and diabetic kidney disease [119].

In addition to the above studies, quercetin has been shown to have other specific clinical effects. After eight weeks of quercetin treatment (500 mg/day) in women with rheumatoid arthritis, early morning stiffness (EMS), morning, and after-activity pain were reduced, and TNF-α levels were significantly decreased [120]. Eight weeks of quercetin supplementation at 500 mg/day in post-myocardial infarction patients was shown to significantly increase TAC and improve quality of life (QOL) [121]. Quercetin also had an effect on polycystic ovary syndrome (PCOS), with lower plasma levels of resistin, testosterone, and luteinizing hormone (LH), higher levels of adiponectin, and improved insulin resistance in patients treated with quercetin compared to those treated with placebo [122,123]. In addition, other clinical studies have demonstrated the beneficial effects of quercetin in the treatment of β-thalassemia [124], recurrent urinary tract infections [125], hemorrhoidal disease [126], sarcoidosis [127], diabetic foot ulcers [128], and chronic hepatitis C [129].

### 4.2. The Application Prospects and Limitations of Quercetin

Taking the above clinical studies together, we can confirm that quercetin does improve human health in other diseases with promising applications, although some of the mechanisms of action are still unclear. So far, there is no clinical trial to support that quercetin can also improve testicular function in human male infertility patients; quercetin might have good clinical application potential based on its pharmacological activity. In fact, there are already many extracts and nutraceuticals of quercetin and its derivatives on the market, and once their efficacy is better clinically validated, they will be more widely used as complementary therapeutic tools in the treatment of a variety of diseases, playing a role in promoting human health.

However, it is important to note that the use of quercetin is also limited by many factors and its effect on certain physiological processes in the body is somewhat controversial. Although quercetin has been shown to ameliorate premature ovarian failure (POF) [130,131] in mice, it has also been reported that quercetin may cause ototoxicity, and female *Oryzias latipes* exposed to excess quercetin for six weeks were found to have a slight increase in TUNEL-positive cells in the intestine, liver, kidney, and ovaries, as well as an increase in ovarian follicular atresia [132]. Because quercetin is an aromatase inhibitor in humans, fish, and other organisms, its inhibition of estrogen production may explain this phenomenon. Furthermore, among all the pharmacological properties of quercetin, the antioxidant capacity is the most prominent and significant one, and is the main mechanism that plays a role in combating testicular damage and improving the symptoms of various diseases, but different results have been reported as well. Ranawat et al. showed that quercetin is also a pro-oxidant, and when mice were injected intraperitoneally with quercetin at different concentrations (2, 8 and 20 mg/kg body weight), testicular levels of ROS, MDA showed a dose-dependent increase, while CAT, SOD, and GSH showed a dose-dependent decrease; moreover, quercetin disrupted sperm concentration, viability, and seminiferous tubule morphology [133], suggesting that more in-depth experiments are needed to validate the appropriate dose of quercetin application.

Interestingly, there is an important but extremely overlooked issue in the practical application of quercetin for the treatment of male infertility, and that is whether or not quercetin will interact with other drugs, nutraceuticals, or even microorganisms, which can cause unknown effects on the organism. This is especially true for the gut microbiome that resides in the human gut, which is closely related to individual human health. Recent studies also suggest that the gut microbiome plays a role in human reproductive health. Immune activation caused by gut microbiome translocation leads to testicular and epididymal inflammation and ultimately affects spermatogenesis, and it also has an impact on sex hormones and sexual function [134]. Many drugs and chemicals also cause testicular damage by inducing gut microbiota dysbiosis, such as glyphosate [135] and BPA [136], although the mechanism remains to be further studied. In contrast, probiotics targeting the gut microbiome have been shown to have beneficial effects on male reproduction, improving sperm motility and viability parameters [137], as well as reducing inflammation and oxidative stress induced by diethylhexylphthalate [138]. The mechanism by which some drugs or natural compounds (cyanidin-3-O-glucoside and catalpol) ameliorate testicular damage also seems to be related to the regulation of the gut microbial community [139,140]. Based on the study mentioned above, whether quercetin and the gut microbiome can interact with each other is also a topic worth exploring, and its elucidation will help us better understand the effects of quercetin on the testis.

It is well-documented that combining drugs is a good therapeutic option, and besides quercetin, several other drugs and natural products have been shown to have ameliorative effects on testicular damage. Portulaca oleracea, an annual herbaceous plant, showed testicular protection in Streptozotocin-induced type 1 diabetic rats, mainly in terms of attenuation of oxidative stress and improvement of sperm parameters [141]; a plantain-based diet also showed improvement of oxidative stress and inflammation levels in the testes of rats after atrazine exposure [142]; resveratrol enhanced testicular antioxidant activity and attenuated iron-exposure-induced sperm quality impairment [143] and testicular apoptosis in mice with type 1 diabetes [144], etc. The benefits of quercetin in combination with sitagliptin have been demonstrated [99], but whether the above or other drugs and natural products in combination with quercetin will have a better effect on testicular damage is still unknown and needs to be explored in further studies.

How to get quercetin or related products into the human body in an uncomplicated way and ensure its action is also a problem to be overcome in clinical applications. Although it is unfortunate to find that no studies have explored whether quercetin can cross the BTB, and the concentration of quercetin in testicular tissue remains unknown, there is no doubt that the health-promoting effects of quercetin can only be achieved when the plasma concentration of quercetin reaches above effective levels. Unfortunately, when quercetin-rich foods are ingested by the body, it interacts with digestive juices such as saliva during digestion and absorption into the bloodstream, and may also be degraded to phenolic acid under the highly acidic conditions of the stomach, or glucuronidation or methylation under the action of various enzymes in the intestine [21]; in addition, the metabolism of quercetin is also rapid [145], and these factors indicate that the effective concentration of quercetin in plasma does not reach a dose sufficient to perform its function, meaning that the oral utilization of quercetin is not high, which will greatly limit its therapeutic effect. When this deficiency is compensated by increasing the dose, it is not safe enough for humans. At present, lipid-based systems have been widely used as drug carriers, and quercetin as its carrier has been produced for many years, and its improved bioavailability and efficacy for quercetin have been confirmed; nanoparticles, nanocrystals, and polymers such as hydrogel beads have also been used for oral delivery of quercetin [21,145], and the use of these technologies plays an important role in enhancing the value of quercetin as a functional food. In addition to improving the oral utilization of quercetin, another extremely important question is what is the appropriate dose of quercetin to administer. There are no universally used doses in clinical trials of quercetin, and the doses of quercetin used in studies for different populations and study purposes vary widely. Importantly, the doses used in rodents do not have sufficient reference value. Thus, it seems that further targeted studies on the dosage of quercetin use and thorough experiments to clarify its safety for long-term use as a dietary supplement are an indispensable step to realize the health benefits of quercetin and promote human health [21].

## 5. Conclusions

Overall, the studies reviewed here clearly indicate the potential of quercetin to protect testis from damage. In fact, quercetin has been proven to have many physiological effects and biological activities, achieving a variety of positive effects such as antioxidant, anti-apoptotic, anti-cancer, and anti-inflammatory—with undeniable health-promoting properties—and is a very promising health supplement. In this review, we have summarized the possible mechanisms through which quercetin may be mediating these effects. Additionally, we focused on the potential benefits of quercetin on male testicular damage, which is a major cause of male infertility due to adverse lifestyle, chronic metabolic diseases, occupational exposure to environmental pollutants, and cancer treatment. In recent years, flavonoids have become the focus of nutritional research for their efficacy in many pathological conditions, especially quercetin’s exciting ameliorative effect on multifactorial testicular injury, which has been demonstrated in a wide range of animal experiments. Although its preventive and protective effects on human male infertility patients need to be further confirmed by epidemiological studies including clinical trials, it is still possible to maintain an optimistic attitude and actively resolve the difficulties of extraction and use.

## Figures and Tables

**Table 1:** The protective effects of quercetin on exposure to different environmental pollutants.

Types of Pollutants		Exposure Time and Dose	Animal Model	Quercetin Dose and Duration	The Effect of Quercetin	Reference
Heavy metals	Pb	PbAc: 50 mg/kg bw/d, oral for 60 days Al_2_O_3_NPs: 100 mg/kg bw/d, oral for 60 days	SD rats	20 mg/kg bw/d, oral for 60 days	reversed adverse effects on testis weight, improved sperm parameters, increased serum testosterone level, increased antioxidant enzymes and decreased malondialdehyde (MDA), improved histology, decreased pro-inflammatory cytokines.	[71]
		150 mg/kg bw/d, gavage for 34.5 days (one cycle of the seminiferous epithelium)	NMRI strain male mice	75 mg/kg bw/d, gavage for 34.5 days	restored morphological changes, increased number of testicular cells, reduced apoptosis level.	[75]
		150 mg/kg bw/d, oral gavage for at least 35 days	NMRI strain male mice	75 mg/kg bw/d, oral gavage for at least 35 days	increased the expression of connexin 43 (C × 43).	[76]
	Cd	5 mg/kg bw/d, oral for 4 weeks	Wistar rats	20 mg/kg bw/d, oral for 4 weeks	improved testis weight, recovered semen quality and serum reproductive hormones, decreased glucose, lactate, and lactate dehydrogenase (LDH) in testis, increased enzymatic and non-enzymatic antioxidants level.	[68]
		15 mg/kg bw/d, oral for 5 days	Wistar rats	10 mg/kg bw/d, oral for 8 days (including 3 days of pretreatment)	improved the sperm characteristics, increased plasma hormone level, reversed superoxide dismutase (SOD), catalase (CAT), glutathione peroxidase (GPx), and glutathione S-transferase (GST) activities and restored the H_2_O_2_ and MDA levels, attenuated histological damage.	[72]
		4 mg/kg bw/d, oral for 2 weeks	ICR mice	75 mg/kg bw/d, oral for 2 weeks	enhanced antioxidant capacity, inhibited cell apoptosis.	[73]
		2 mg/kg bw/d, intraperitoneal injection for 4 weeks	SD rats	50 mg/kg bw/d, intragastrical administration for 4 weeks	reversed bad effects on testis weight and body weight, relieved oxidative stress status, alleviated histopathological disorder, ameliorated the expression of P62 and LC3-II.	[78]
		5 mg/kg bw/d, oral for 4 weeks	Wistar rats	20 mg/kg bw/d, oral for 4 weeks	reversed adverse effect to sexual behavior, downregulated serum NO concentration and testicular cholesterol concentration, recovered 3β-hydroxysteroid dehydrogenase (3β-HSD) and 17β-HSD activity, increased testicular testosterone concentration.	[79]
	Mn	15 mg/kg bw/d, oral for 45 days	Wistar rats	10 or 20 mg/kg bw/d, oral for 45 days	increased antioxidant enzyme activities, decreased inflammatory biomarkers and cleaved caspase-3 in the brain, testes, and epididymis, modulated circulatory hormones concentration and marker enzymes of testicular function, augmented sperm functional parameters, prevented histological changes.	[80]
	Zn	100 or 400 mg/kg bw/d, intragastric intubation for 12 weeks	Albino rats	100 mg/kg bw/d, intragastric intubation for 12 weeks	improved sperm parameters, ameliorated oxidative stress, restored testosterone level and steroidogenesis, improved histology.	[81]
	Ti	300 mg/kg bw/d, oral gavage for 35 days	NMRI mice	75 mg/kg bw/d, oral gavage for 42 days	increased testis weight, increased serum testosterone level and testosterone concentration in testis, increased sperm count and motility, attenuated histopathological damage, decreased apoptotic index, improved SOD and CAT activities, attenuated MDA activities.	[82]
Other environmental pollutants	Atrazine	232 μM for 6 h	Wistar ratsLeydig cells	50 μM for 6 h	normalized the expressions of steroidogenesis genes.	[83]
		50 μg/mL for 6 or 24 h	Wistar ratsLeydig cells	50 μM 6 or 24 h	recovered cell viability, improved oxidative stress and lipid peroxidation, prevented the activities of steroidogenesis enzymes, restored nuclear factor kappa-B (NF-κB) mRNA and protein levels.	[84]
		232 μM for 6 or 24 h	Wistar rats Sertoli cells	50 μM for 6 or 24 h	improved cell viability, attenuated oxidative damage; upregulated SOD-1, GPx, glutathione reductase (GR), and GST expressions.	[69]
		120 mg/kg bw/d, oral gavage for 21 days	Albino rats	10–50 mg/kg bw/d, oral gavage for 21 days	increased the body weight, improved the antioxidant capacity, restored serum testosterone and sperm morphology, increased the serum IgA, inhibited the percentage of DNA fragmentation, disturbed the cytochrome P450 family 17 subfamily A member 1 (*Cyp17a1*) mRNA expression, slightly improved histology.	[85]
		50 mg/kg bw/d, oral every other day for 60 days.	Wistar rats	5–10 mg/kg bw/d, oral every other day for 60 days.	reduced GSH and GST concentrations, decreased MDA concentrations, enhanced anti-inflammatory effects, reversed serum hormones level, increased morphometric parameters and sperm quality parameters, improved histopathology.	[86]
	As	10 mg/kg bw/d, oral gavage for 15 days	SD rats	50 mg/kg bw/d, oral gavage for 15 days	lessened terminal dUTP nick end-labeling (TUNEL)-positive germ cells, improved proliferating cell nuclear antigen (PCNA)-positive cells, amplified antioxidant effect.	[87]
		50 ppm in drinking water for 49 days	SD rats	50 mg/kg bw/d, oral gavage for 49 days	ameliorated histology and morphometric, increased antioxidant enzymes and testosterone level in testis and plasma.	[88]
	BPA	80–240 mg/kg bw/d, oral gavage for 45 days	Swiss strain albino mice	30–90 mg/kg bw/d, oral gavage for 45 days	increased body weight and testis weight, increased serum testosterone level and the activity of steroidogenic enzymes.	[89]
	COV	inhalation for 5 h daily in pollution chamber for 30 days	Wistar rats	50 mg/kg bw/d, oral for 30 days	improved testicular weight, gonadosomatic index and sperm parameters, alleviated histopathological damage, reduced apoptotic, increased antioxidant capacity.	[90]
	DEPs	220 μg/mouse, subcutaneous injection for 10 times during 5 weeks	BALB/cmice	Feed with 0.3%, 0.1%, 0.03% *w*/*w* quercetin, oral for 30 days in CE-2 commercial diets	restored spermatogenesis and sperm morphological abnormalities, increased the numbers of Sertoli cells.	[91]
	PCBs	2 mg/kg bw/d, intraperitoneal injection for 25 days	Wistar rats	50 mg/kg bw/d, oral for 25 days	restored oxidative stress indices, reversed DNA fragmentation, reversed histological damage.	[92]
	CCl_4_	0.25 mL/kg bw/w, oral gavage for 10 weeks	Wistar rats	150 mg/kg bw/d, oral gavage for 10 weeks	decreased MDA level, improved abnormal sperm rate, reduced histopathological lesions and apoptosis in testis.	[93]
	PNP	50 mg/kg bw/d, intraperitoneal injection for 6 weeks	ICR mice	75 mg/kg bw/d, intraperitoneal injection for 6 weeks	attenuated histopathological damage; changed antioxidant status caspase-3 activity and number of TUNEL-positive cells; altered BCL-2 associated X protein (BAX), B-cell lymphoma-extra large (BCL-XL), X Box binding protein (XBP-1), and heme oxygenase-1 (HO-1) expression.	[94]
	TCDD	2 μg/kg bw/w, oral gavage for 60 days	Wistar rats	20 mg/kg bw/d, oral gavage for 60 days	increased antioxidant capacity, increased sperm parameters and testis weight, reversed histopathological changes, increased serum testosterone levels.	[95]
	acetylene	inhalation 20 min daily for 30 days in pollution chamber with 58,000 ppm acetylene	Wistar rats	30 mg/kg bw/d for 30 days	improved biochemical indexes and histopathological damage.	[96]
	PEs	900 mg/kg bw/d, oral for 30 days	SD rats	10, 30 and 90 mg/kg bw/d, oral for 30 days	increased testis weight and epididymis weight, increased serum testosterone, luteinizing hormone (LH), follicle-stimulating hormone (FSH), and estradiol level, inhibited testicular injuries, downregulated steroidogenic proteins expression, restored spermatogenesis.	[97]
		300, 600, or 900 mg/kg bw/d, oral gavage for 15 days	Wistar rats	90 mg/kg bw/d, 24 h before DEHP treatment	improved relative testes weight and sperm parameters, increased serum testosterone and prostatic acid phosphatase, reverted antioxidant enzyme activities, ameliorated histologic alterations.	[98]

## Data Availability

Not applicable.
